# MSC secreted extracellular vesicles carrying TGF-beta upregulate Smad 6 expression and promote the regrowth of neurons in spinal cord injured rats

**DOI:** 10.1007/s12015-021-10219-6

**Published:** 2021-08-27

**Authors:** Tianyu Han, Peiwen Song, Zuomeng Wu, Xia Xiang, Yunlei Liu, Ying Wang, Huang Fang, Yang Niu, Cailiang Shen

**Affiliations:** 1 Department of Orthopedics (Spinal Surgery), The First Affiliated Hospital of Anhui Medical University, 218 Jixi Road, Shushan District, Hefei City, Anhui Province China; 2 Department of Clinical Laboratory, The NO.2 People’s Hospital, Fuyang City, Fuyang China; 3 Department of Medical Imaging, The First Affiliated Hospital of Anhui Medical University, Hefei, China; 4 Department of Spinal Surgery, The First Affiliated Hospital of USTC, Hefei, China

**Keywords:** Mesenchymal stem cells, Transforming growth factor β, Smad 6, Neural stem cells, Spinal cord injury

## Abstract

Mesenchymal stem cells (MSCs) constitute a promising therapy for spinal cord injury (SCI) because they can provide a favorable environment for the regrowth of neurons by inhibiting receptor-regulated Smads (R-Smads) expression in endogenous neural stem cells (NSCs). However, their mechanism of action and effect on the expression of inhibitory Smads (I-Smads) remain unclear. Herein, we demonstrated that extracellular vesicles (EVs) from MSCs were able to upregulate the Smad 6 expression by carrying TGF-β, and the Smad 6 knockdown in NSCs partially weakened the bone marrow MSC (BMSC)-EV-induced effect on neural differentiation. We found that the expression of Smad 6 did not reduced owing to the TGF-β type I receptor kinase inhibitor, SB 431,542, treatment in the acute phase of injury in rats with SCI, thereby indicating that the Smad 6 expression was not only mediated by TGF-β, but also by the inflammatory factors and bone morphogenetic proteins (BMPs) as well. However, in the later phase of SCI, the Smad 6 expression decreased by the addition of SB 431,542, suggesting that TGF-β plays a key role in the mediation of Smad 6 expression in this phase. In addition, immunohistochemistry staining; hematoxylin–eosin staining; and the Basso, Beattie, and Bresnahan (BBB) scores revealed that the early inhibition of TGF-β did not increase neuron regrowth. However, this inhibition increased the cavity and the caspase-3 expression at 24 h post-injury, leading to a worse functional outcome. Conversely, the later treatment with the TGF-β inhibitor promoted the regrowth of neurons around the cavity, resulting in a better neurological outcome. Together, these results indicate that Smad 6 acts as a feedback regulator to prevent the over-differentiation of NSCs to astrocytes and that BMSC-EVs can upregulate Smad 6 expression by carrying TGF-β.

**Graphical abstract:**

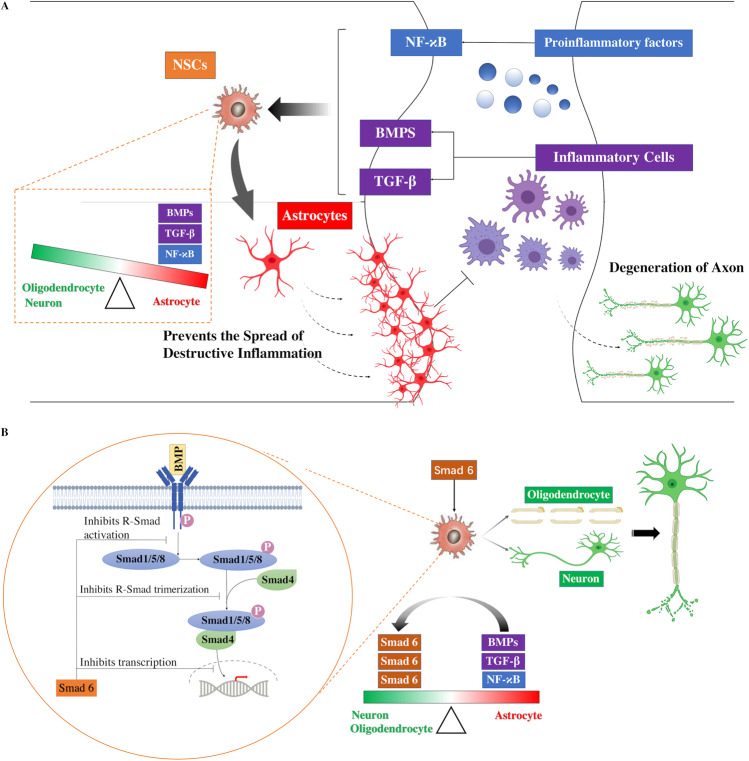

## Introduction

Spinal cord injury (SCI) is often caused by primary mechanical injury to the spinal cord, followed by a series of molecular and cellular interactions. It results in necrosis, degeneration, and the demyelination of axons, as well as neuronal apoptosis, which results in the permanent impairment of neurological functions [1, 2]. In the early phase of injury, endogenous neural stem cells (eNSCs) are spontaneously activated and migrate into the injured cores [3]. These activated eNSCs have long been thought to assist in self-recovery by replacing lost nerve cells [4, 5]. However, emerging studies have found that most of these cells differentiate into astrocytes rather than into neurons and oligodendrocytes [3]. Glial scars, which consist mainly of astrocytes, have proven to be advantageous in limiting the spread of inflammation in the acute phase of SCI, thereby protecting the surviving nerve cells around the injured lesion [6–8]. In spite of this, excessive scar growth around the injured lesion keeps the neural circuit from reorganizing [9].

Smads, which are ligand-activated receptors, have been shown to be closely associated with scar formation. They can be directly induced by members of the transforming growth factor β (TGF-β) family and are classified into three types: receptor-activated Smads (R-Smads), which include Smad 1/5/8 and Smad 2/3; the common Smad (co-Smad), Smad 4; and inhibitory Smads (I-Smads), Smad 6 and Smad 7 [10]. In response to ligand stimulation, R-Smads form a heterotrimeric complex with Smad 4. These complexes are then translocated to the nucleus and induce the expression of a number of genes [11], which in turn promotes astroglial generation [5, 11, 12]. I-Smads, which are primarily localized in the nucleus of most cells, can be upregulated by TGF-β, bone morphogenetic proteins (BMPs), UV irradiation, and some pro-inflammatory cytokines [13–17]. Activated I-Smads function as transcriptional regulators in the nucleus and inhibit intracellular activation by interacting with R-Smads. Smad 6, one of the I-Smads, binds directly to BMP type I receptors and prevents the downstream phosphorylation of Smads by BMP. Smad 6 has also been shown to form a complex with activated Smad 1, while it prevents the latter from forming a complex with Smad 4 [18, 19]. In addition, Smad 6 could potentially accelerate the degradation of BMP-induced Smads. Evidence has shown that Smad 6 recruits Smurf1, which forms a complex with BMP-induced Smads and enhances their degradation[19]. Furthermore, Smad 6, together with histone deacetylases and transcription factors, interferes with BMP/Smads-induced gene expression [20, 21]. Through these mechanisms, Smad 6, when activated by BMP or TGF-β, acts as a negative feedback regulator in TGF-β superfamily mediated signaling. Therefore, the upregulation of I-Smads is considered to be effective in preventing excessive glial scar formation.

The transplantation of mesenchymal stem cells (MSCs) is a promising therapy for SCI [22], as MSCs, which were first isolated from bone marrow [23], can differentiate into three main types of nerve cells. Researchers have transplanted MSCs into injured spinal cords in an attempt to promote their differentiation into neurons and oligodendrocytes with the goal of replacing lost nerve cells [24, 25]. However, emerging evidence suggests that MSCs promote neurological recovery by providing a favorable environment for axon regrowth and protecting surviving nerve cells from apoptosis, rather than by directly replacing the lost nerve cells [26]. Recent studies indicate that MSCs inhibit Smad 1/5/8 phosphorylation, which, in turn, prevents the overgrowth of glial scars. However, few studies have been conducted to assess whether MSCs can regulate the expression of I-Smads, which mediate the action of R-Smads and the differentiation of NSCs in SCI [5].

This study focused on bone marrow mesenchymal stem cells (BMSCs) and their possible effects on the regulation of Smad 6 expression. We established that BMSC-extracellular vesicles (BMSC-EVs) were able to upregulate Smad 6 expression in NSCs. Blocking TGF-β diminished the BMSC-EVs -related upregulation of Smad 6 expression, thereby suggesting that BMSC-EVs mediated Smad 6 expression through the secretion of TGF-β. Moreover, Smad 6 knockdown in NSCs partially weakened the BMSC-EVs-mediated effect on the neural differentiation of NSCs, thereby indicating that Smad 6 may act as a negative regulator to prevent the overproduction of astrocytes. Additionally, the addition of the TGF-β type I receptor kinase inhibitor to BMSC-EVs-treated rats reduced the Smad 6 expression only in the later phase of injury, thereby indicating that upregulation of Smad 6 was closely associated with BMSC-EVs treatment in SCI rats.

## Methods

### Culture, differentiation, and transfection of NSCs

NSCs were cultured as previously described in our previous studies [27, 28]. The cells were obtained from the subventricular zone of the SD rats. The isolated cells were cultured as suspended neurospheres in DMEM/F12 (Gibco, USA) using 20 ng/mL epidermal growth factor (EGF) (Gibco, USA), 2% B27 (Gibco, USA), and 10 ng/mL basic fibroblast growth factor (bFGF) (Gibco, USA) for seven days. The medium was changed every three days.

To knockdown Smad 6 in NSCs, siRNAs (sense, 5′-GAUUCUACAUUGUCUUACA-3′; antisense, 5′-UGUAAGACAAUGUAGAAUC-3′) were transfected into passage 2 NSCs using Lipofectamine 2000 (Invitrogen) for 24 h. PCR was used to confirm the effect of Smad 6 knockdown in NSCs. Non-targeting siRNA was used as a negative control.

Passage 2 NSCs or the Smad 6-knockdown NSCs were dissociated and reseeded on glass coverslips in 5% FBS-DMEM/F12 for 24 h. The medium was then switched to DMEM/F12 supplemented with one of the following: BMSC- EVs or 10 ng/mL TGF-β (R&D Systems); BMSC-EVs + 10 μM SB431542 [the TGF-β type I receptor kinase inhibitor (Sigma)]; BMSC-EVs + 20 ng BMP4; 10 ng/mL TGF-β + 10 μM SB431542; 10 ng/mL TGF-β + 20 ng BMP4 (R&D Systems); 20 ng/mL IL-6 (Sigma), with or without 30 μM JSH-23 (NF-κB inhibitor, MCE); 20 ng/mL IL-6 + BMSC-EVs, with or without SB 431,542; 20 ng/mL BMP4, with or without 200 ng/mL Noggin [BMP- antagonist (Sigma)]; 20 ng/mL BMP4 + BMSC-EVs, with or without SB 431,542; 40 ng/mL IL-6 and 40 ng/mL BMP4, with or without BMSC-EVs. The medium was changed every three days. The cells were cultured for 7 days and then subjected to immunohistochemistry and protein collection.

### Mesenchymal stem cell culture and the preparation of BMSC-EVs

MSCs were cultured as described in our previous studies [27, 28]. The cells were isolated from the bone marrow of Fischer 344 rats and cultured in DMEM (low glucose, Hyclone) containing 10% fetal bovine serum (FBS) (Gibco, USA) and 1% antibiotic solution at a density of 1 × 10^6^ cells/cm^2^. The medium was removed, along with non-adherent cells, after 24 h of culture. The residual adherent cells were reseeded at a density of 8,000 cells/cm^2^ in 10% FBS-DMEM. The medium was changed every three days and was passaged when 90% confluence was reached.

When the passage 3 BMSCs reached a 90% confluence, we collected the supernatant as described in previous studies [27, 29]). To remove the cell debris, the collected conditioned medium was centrifuged at 300 × g for 10 min, then at 2,000 g for 20 min, and finally at 10,000 × g for 30 min at 4 °C. Next, BMSC-EVs were collected by a centrifugation at 10,0000 g for 60 min at 4 °C. Transmission electron microscopy (TEM) and western blot analysis (the antibodies used were as follows: 1:1000 CD 63, 1:2000 CD 9, 1:1000 TSG 101, and 1:1000 CD 90), and detection of the diameter of BMSC- EVs (by dynamic light scattering) were used to identify BMSC-EVs. The harvested BMSC-EVs were then dissolved in 100 μL of PBS and stored at − 80 °C.

### ELISA

The level of TGF-β in BMSC-EVs was determined using an ELISA kit (Sigma) according to the manufacturer’s protocol.

### Animal protocols (spinal cord treatment)

Animal procedures were approved by the Ethics Committee of Anhui Medical University (No. 20191064), in accordance with the guidelines of the Declaration of Helsinki, revised in Edinburgh in 2000. Details of the procedure have been outlined in our previous study [27]. To briefly summarize, a laminectomy was performed at the T10 level in female Wistar rats (6–8 weeks old, weighing 200–250 g). Rats were randomly divided into sham, SCI (control, treated with DMEM/F12), BMSC-EVs- treated, BMSC-EVs + SB431542_day0_-treated, and BMSC-EVs + SB431542_day3_-treated groups. Power analysis was performed to determine the sample sizes. An infinite horizons spinal cord impactor (IH-0400) was used to induce a direct weight drop injury. A mini osmotic pump (Alzet 1007D, USA) filled with either DMEM/F12 (control), BMSC-EVs, or BMSC-EVs + SB431542_day0_ was linked to a soft catheter and implanted under the dura. The medium in the pump was released at a rate of 1 μL/h, and the pump was removed after three days (for details, refer to the manufacturer’s protocols and Franzen et al. [29]). For the BMSC-EVs + the SB431542_day3_-treated groups, we injected SB431542 through the residual catheter after removing the pump on day 3 following the onset of SCI. The motor function of the lower extremities of SCI rats was evaluated blindly by two independent individuals according to the Basso, Beattie, and Bresnahan (BBB) open-field test[30] at ldifferent time points (days 1, 4, 7, 14, 17, 21, 24, and 28).

### Tissue processing and immunofluorescence staining

Spinal cords were removed from 4-week post trauma rats and fixed in 4% paraformaldehyde for 30 min. A 3-mm long section of the spinal cord, centered on the injured area, was cut into 35-μm-thick sections using a Leica RM2135 microtome. The sections were prepared for immunohistochemistry as described in our previous studiy [31]. The primary antibodies used included mouse anti-Map-2 for neurons (1:500; Abcam, UK) and rabbit anti-glial fibrillary acidic protein (GFAP) for astroglia (1:1000; Abcam, UK); the secondary antibodies used were Alexa Fluor 488 (green, 1:1000; Molecular Probes, Germany) and Cy5 (red, 1:500; Dianova, Germany). The sections were observed and photographed using a DM-6B fluorescence microscope (Leica, Germany) connected to a computer screen.

### RNA extraction and quantitative PCR

Total RNA was extracted from NSCs and tissues using TRIzol (Gibco), according to the manufacturer’s instructions, and cDNA was synthesized using the Superscript III RT Reaction Mix (Invitrogen). Quantitative PCR was performed using the RealPlex2 Mastercycler (Eppendorf) and SYBR Green master mix (Applied Biosystems) with the following cycling parameters: 95 °C for 15 s, and 60 °C for 60 s for 40 cycles. The following gene-specific primers were used: Smad 6: 5′-CTCCGGGTGAATTCTCAGAT-3′, 5′-TGGTCGTACACCGCATAGAG-3`; Id2: 5`-TTTCCTCCTACGAGCAGCAT-3′, 5′-CCAGTTCCTTGAGCTTGGAG-3`; GAPDH: 5`-ACAACTTTGGCATTGTGGAA-3′ and 5′-GATGCAGGGATGATGTTCTG-3′.

### Western blot assay

A 20-mm section of injured spinal cord tissue, centered on the epicenter of the injury, was lyzed in RIPA + PMSF (at a 100:1 ratio of RIPA:PMSF) buffer on ice. The collected proteins were separated by sodium dodecyl sulfate–polyacrylamide gel electrophoresis (SDS-PAGE), transferred to a PVDF membrane, and incubated overnight with primary antibodies at 4 °C (Map-2, 1:2000, Abcam, UK; GFAP, 1:2000, Abcam, UK). This was followed by incubation with a secondary antibody (Santa Cruz Biotechnology; 1:2000 in blocking solution) for 1 h at room temperature. The blots were then visualized using the SuperSignal West Pico enhanced chemiluminescence reagent (Thermo Scientific) and quantified using Image J software.

### Statistical analysis

Data are presented as the mean ± standard error of the mean. Statistical analysis was performed using SPSS software (version 16.0; Chicago, IL, USA). Student’s t-test (two groups) or the one-way analysis of variance (ANOVA) (more than two groups) with Tukey’s post-hoc method were used to test the statistical significance. Statistical significance was set at *p* values < 0.05. For cell counting, 10–15 fields, each containing a total of 500–1000 cells, were randomly selected. The number of positive cells was blindly quantitated by two different individuals.

## Results

### BMSC-EVs affected the expression of Smad 6 in NSCs

**Fig. 1:**
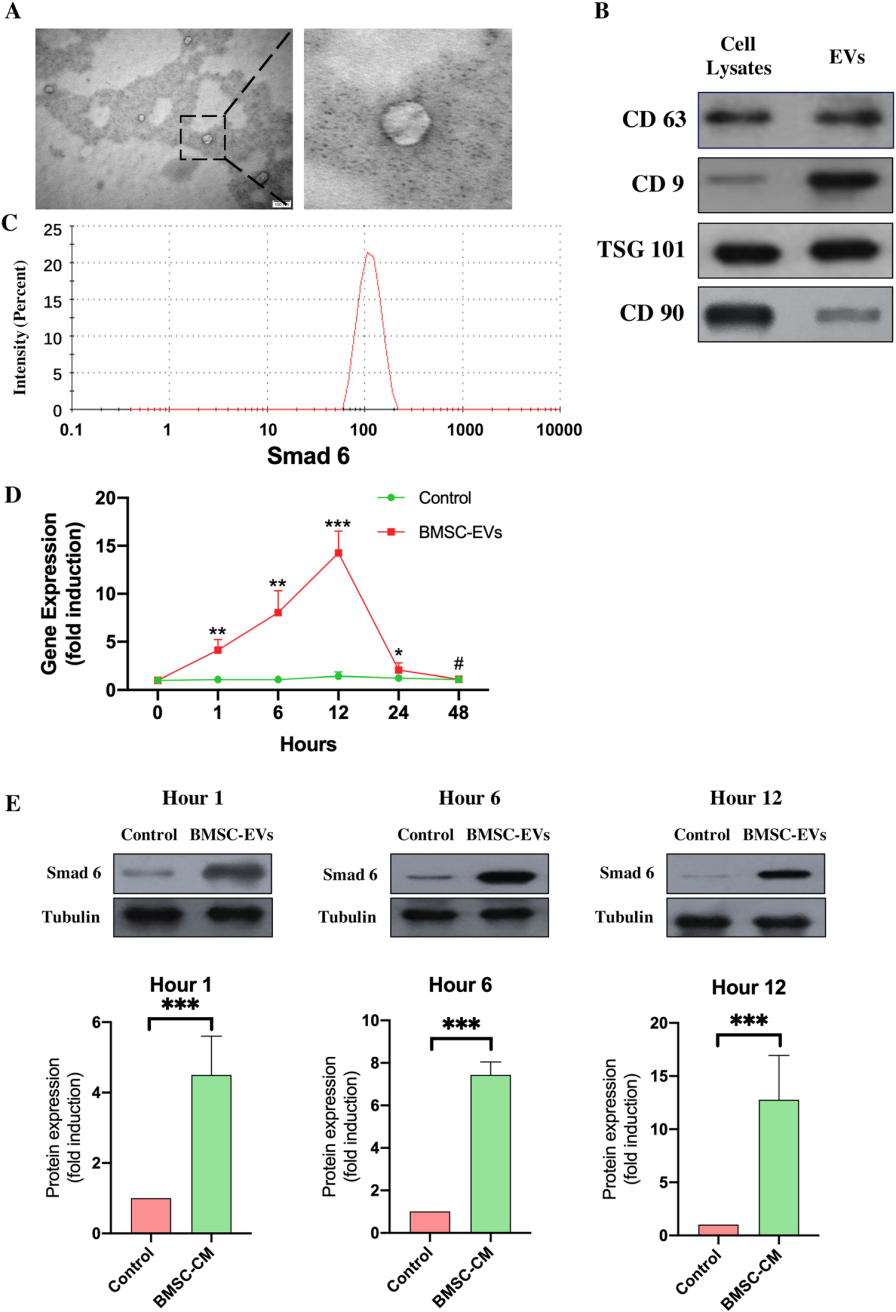
BMSC-EVs upregulate the expression of Smad 6 in NSCs. **A.** Identification of BMSC-EVs by transmission electron microscopy. **B.** Detection of the diameter of BMSC-EVs by dynamic light scattering. **C.** Analysis of CD9, CD63, TSG101, and CD 90 expression by western blot (*n* = 3). **D.** The Smad 6 expression in NSCs increased by the addition of BMSC-EVs and peaked at 12 h (*n* = 5, compared with the control at each time point, Student’s t-test, the data were presented as fold changes to control cells at hour 1). **B.** Western blots analysis confirmed that the Smad 6 expression in NSCs was upregulated by the BMSC-EVs treatment at different time points (*n* = 5, the data were presented as fold changes to control NSCs)

TEM was used to confirm the presence of BMSC-EVs (Fig. 1A), while their diameter of BMSC-EVS was detected by dynamic light scattering (Fig. 1C). The markers of BMSC-EVs were analyzed using western-bloting (Fig. 1B).

To determine whether BMSC-EVs were able to regulate the expression of I-Smads following SCI, we first examined the expression of Smad 6 in NSCs after adding BMSC-EVs. The level of Smad 6 mRNA increased as early as 1 h after BMSC-EVs were added to NSCs. It peaked at 12 h and dropped to the control level at 48 h (Fig. 1D). WB analysis also confirmed this result: NSCs co-cultured with BMSC-EVs had higher expression 1 h, 6 h and 12 h post-co-culture (Fig. 1E). This suggests that Smad 6 expression in NSCs is activated by BMSC-EVs.

### BMSC-EVs altered Smad 6 expression through the secretion of TGF-β

**Fig. 2:**
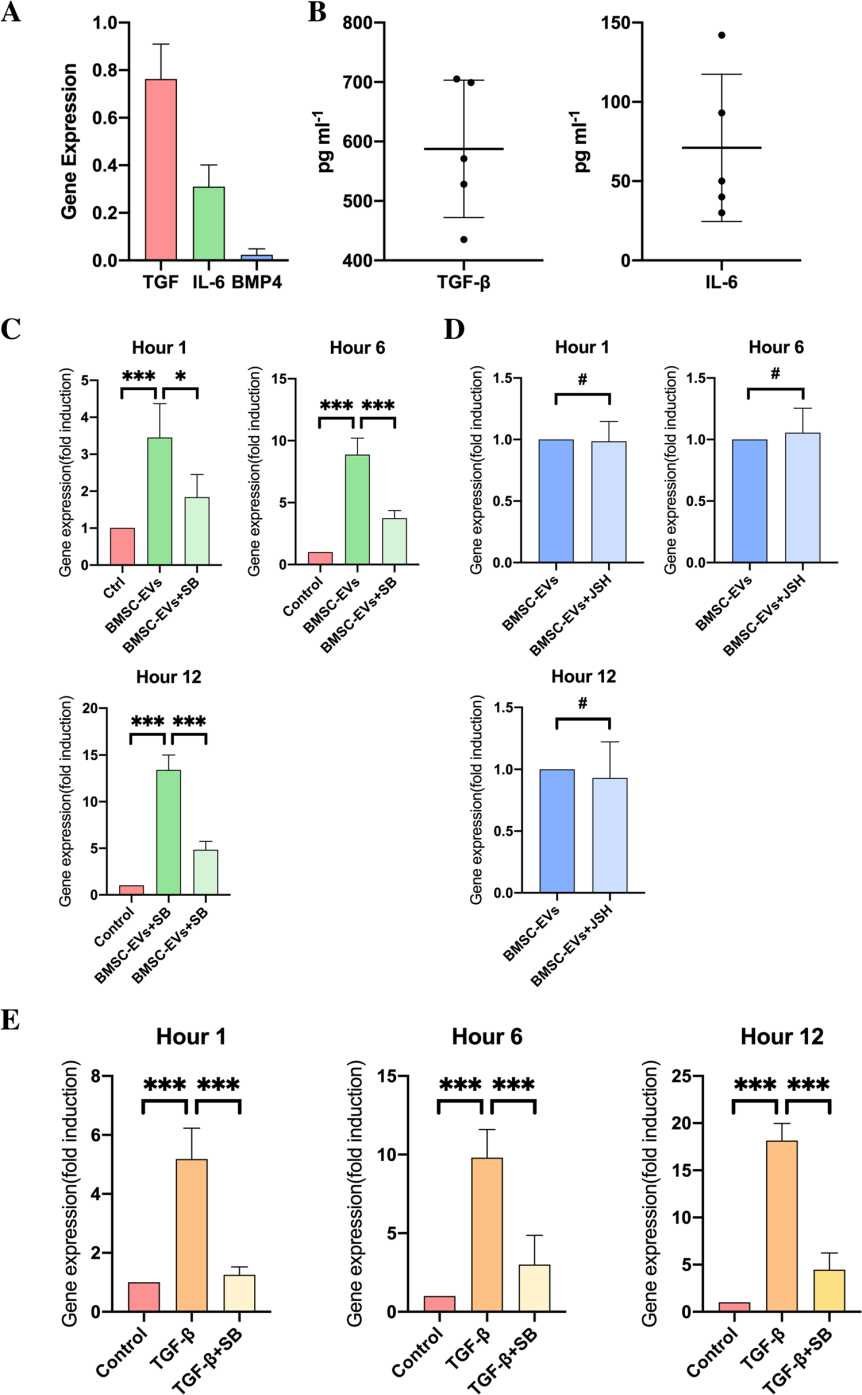
BMSC-EVs upregulated Smad 6 expression in NSCs via the secretion of TGF-β. **A.** To confirm whether IL-6, BMP4, and TGF-β existed in the BMSC-EVs, the expression of these factors was determined by PCR (*n* = 3, Student’s t-test). **B.** ELISA from five individual samples confirmed the presence of TGF-β and IL-6 in BMSC-EVs. **C, D.** To evaluate whether TGF-β or IL-6 played a key role in mediating the expression of Smad 6 in NSCs, we added SB431542 and JSH-23, respectively, to NSCs in the presence of BMSC-EVs. This revealed that the BMSC-EVs-induced upregulation of Smad 6 could be abolished by the addition of SB431542 (C, n = 5; the data were revealed as fold changes to control NSCs, Student’s t-test). Conversely, the addition of JSH-23 did not alter the expression of Smad 6 in NSCs (D, n = 5; the data were presented as fold changes to control BMSC-EV treated NSCs, Student’s t-test) **E.** We added TGF-β to NSCs to assess the effect of TGF-β on mediating Smad 6 expression; this resulted in an increase of Smad 6 expression in NSCs. This TGF-β-induced upregulation of Smad 6 expression could be significantly abolished by the addition of SB431542 (*n* = 5; the data were presented as fold changes to control NSCs, Student’s t-test). (Smad 6 mRNA expression was normalized to GAPDH mRNA; the data were presented as mean ± S.D; ^*^p < 0.05, ^**^p < 0.01, ^***^p < 0.001, ^#^*p* > 0.05.)

Previous studies have reported that BMPs, TGF-β, and NF-κB signaling can upregulate the Smad 6 expression [13–17]. We first used RT-PCR to determine whether BMP4, TGF-β, and IL-6 (which was proven to be able to activate NF-κB signaling during the inflammatory process) were present in the BMSC-EVs by RT-PCR. The results confirmed that only TGF-β and IL-6 were present in the BMSC-EVs, and the expression of BMP4 was not observed (Fig. 2A). We then measured the concentrations of TGF-β and IL-6 in BMSC-EVs using ELISA. The ELISA assays of all five of BMSC-EVs samples showed that TGF-β had an average concentration of approximately 590 (587 ± 115) pg/mL and IL-6 had a concentration of 70 (71 ± 46) pg/mL (Fig. 2B).

To further determine whether the BMSC-EVs-induced upregulation of Smad 6 expression was associated with TGF-β and IL-6, we added the TGF-β type I receptor kinase inhibitor (SB431542) and NF-κB inhibitor (JSH-23) to the NSCs in the presence of BMSC-EVs to evaluate whether the Smad 6 expression in NSCs could be reduced by the addition of these inhibitors at different time points. The results revealed that the BMSC-EVs induced upregulation of Smad 6 in NSCs was suppressed by the addition of SB431542 (Fig. 2C). In contrast, the addition of JSH-23 to NSCs did not alter Smad 6 expression in the presence of BMSC-EVs (Fig. 2D), indicating that TGF-β secreted by BMSCs played a key role in elevating the Smad 6 expression in NSCs. To further determine whether TGF-β was able to regulate Smad 6 expression in NSCs, we examined Smad 6 mRNA levels in the TGF-β-treated NSCs at different time points by using RT-PCR and found that the addition of TGF-β markedly increased Smad 6 expression in NSCs. Moreover, this TGF-β-induced alteration could be nullified by the TGF-β type I receptor kinase inhibitor SB431542 (Fig. 0.2E).

Studies have reported that Smad 6 is regulated in response to various factors, including BMP, TGF-β, and NF-κB signaling. BMSCs have been shown to be a negative regulators of BMP-Smad 1/5/8 signaling [5] and can repress the production of pro-inflammatory cytokines [32, 33], which are considered to be triggers that activate NF-κB and BMP signaling [34]. Therefore, in agreement with previous results [35],of the three signaling mechanisms, TGF-β acted as a catalysts through which BMSC-EVs regulated Smad 6 expression.

### BMSC-EVs promoted NSC differentiation into neurons partly via the upregulation of Smad 6

**Fig. 3:**
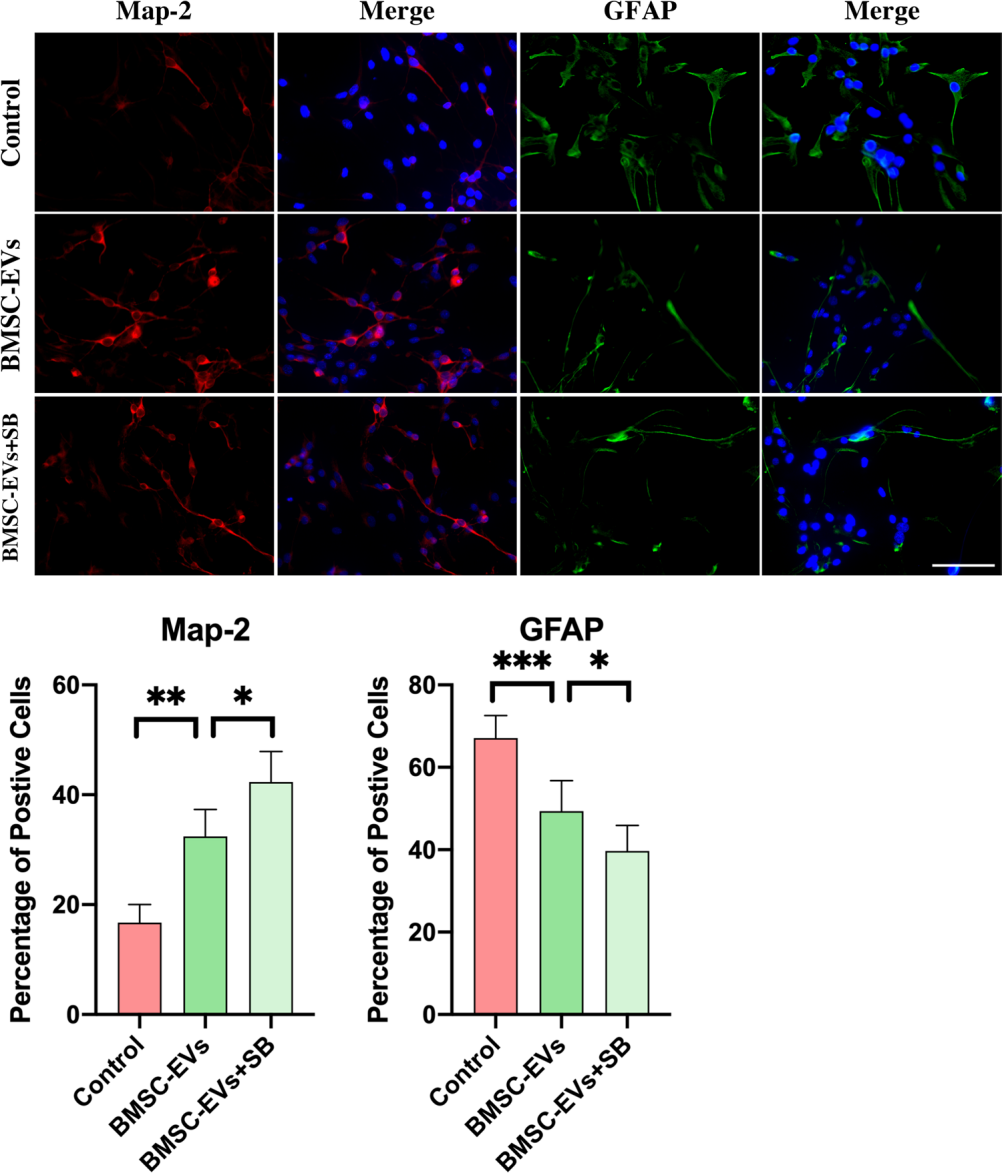
The blocking of TGF-β signaling did not inhibit the differentiation of NSCs into neurons in the presence of BMSC-EVs. To evaluate the effect of BMSC-EVs on the differentiation of NSCs, we added the BMSC-EVs to NSCs and used immunofluorescence to assess the proportion of Map-2^+^ neurons and GFAP^+^ astrocytes. This showed that BMSC-EVs-treated NSCs had a higher proportion of Map-2^+^ cells and a lower proportion of GFAP^+^ cells than NSCs that received only the DMEM/F12 treatment (control group) after 7 days in a co-culture (*n* = 6). However, the addition of SB 431,542 did not decrease the proportion of neurons, which was unexpected. Instead, the proportion of neurons increased; this was accompanied by a reduction in astrocytes by the addition of SB 431,542 in the presence of BMSC-EVs (*n* = 6). Data were shown as mean ± S.D and Student’s t-test was used for comparisons; ^*^p < 0.05, ^**^p < 0.01, ^***^p < 0.001, ^#^*p* > 0.05; scale bars, 100 μm. SB 431,542 is abbreviated as SB

Studies have reported that BMSCs promote the differentiation of NSCs from astrocytes into neurons. Herein, we studied whether Smad 6 was involved in this BMSC-EVs-induced differentiation. Smad 6 acts as a negative feedback regulator in BMP signaling, and therefore, the downregulation of Smad 6 expression can lead to an upregulation of BMP signaling, which should result in an increasing proportion of astrocytes. To downregulate Smad 6 expression, we directly used SB 431,542 to block TGF-β signaling, and the results showed that, compared to the control groups, the exposure of NSCs to BMSC-EVs for 7 days resulted in an increase in microtubule-associated protein 2 (Map-2)-positive neurons and a decrease in glial fibrillary acidic protein (GFAP)-positive astrocytes (Fig. 3). Surprisingly, although the pre-treatment with SB431542 reduced Smad 6 expression, it did not increase the proportion of astrocytes, as expected. Instead, the addition of SB431542 to NSCs in the presence of BMSC-EVs led to a higher proportion of neurons and a lower proportion of astrocytes compared to BMSC-EVs-treated NSCs (Fig. 3). This could be due to the fact that TGF-β itself can inhibit neurogenesis and promote gliosis in the central nervous system (CNS); blocking TGF-β signaling not only reduced Smad 6 expression, but also abolished the TGF-β-induced effect on NSC differentiation. As a feedback regulator, Smad 6 had a weaker mediating effect on the differentiation of NSCs than TGF-β signaling. Therefore, although inhibiting TGF-β signaling had the potential to downregulate Smad 6 expression, it did not lead to the differentiation of NSCs from neurons to astrocytes.

**Fig. 4:**
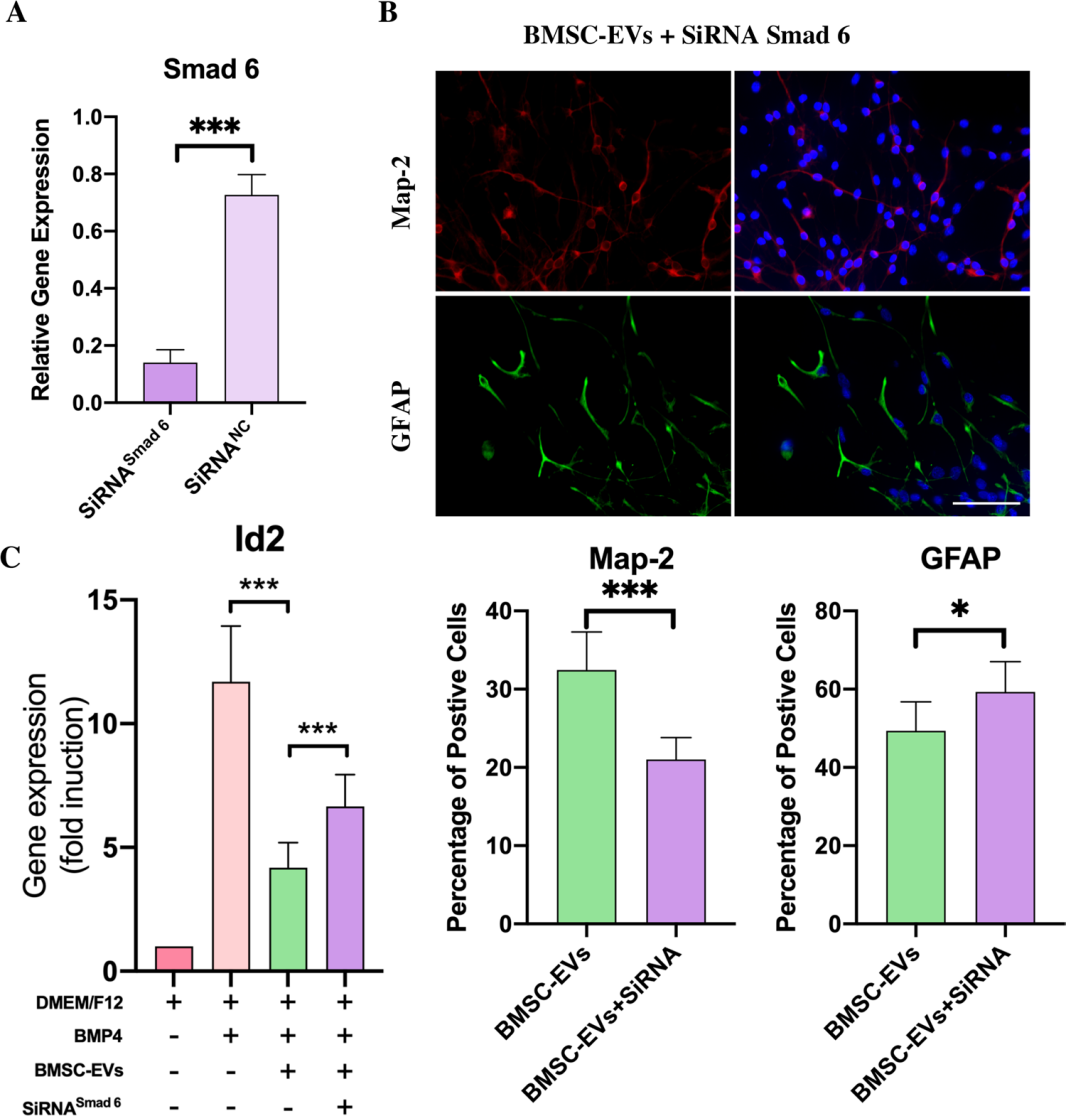
BMSC-EVs promoted the differentiation of NSCs into neurons partly via the upregulation of Smad 6. **A.** PCR results indicated that the expression of Smad 6 in NSCs was markedly reduced by Smad 6 knockdown (*n* = 3). **B.** The BMSC-EVs added to the NSCs, with or without Smad 6 knockdown; the proportion of neurons and astrocytes were calculated by immunofluorescence. This revealed that Smad 6 knockdown partially abolished the BMSC-EVs-induced effect on the differentiation of NSCs, leading to a slightly lower proportion of neurons (*n* = 6). **C.** The treatment of BMP4 on NSCs significantly increased the expression of Id2, and its addition markedly reduced the Id2 expression in the presence of BMP4. Moreover, the Smad 6 knockdown in NSCs somewhat weakened the BMSC-EVs-induced effect on Id2 mediation in the presence of BMP4 (*n* = 5, Id2 mRNA expression was normalized to GAPDH mRNA and the results were revealed as fold changes to control groups). The Data were shown as mean ± S.D; Student’s t-test was used for comparisons, ^*^p < 0.05, ^**^p < 0.01, ^***^p < 0.001, ^#^*p* > 0.05; scale bars, 100 μm

To downregulate Smad 6 expression without inhibiting TGF-β signaling, we used siRNA to knockdown Smad 6 in NSCs, as PCR results indicated that the expression of Smad 6 in NSCs was markedly reduced by Smad 6 knockdown (Fig. 4A). The number of neurons and astrocytes was then calculated using immunofluorescence staining. Compared to the control groups, the addition of BMSC-EVs to Smad 6 knockdown NSCs for 7 days was still able to promote neuronal differentiation (Fig. 4B). However, compared to the NSCs that did not achieve Smad 6 knockdown, the Smad 6 knockdown NSCs had a lower proportion of neurons and a higher proportion of astrocytes after 7 days of being co-cultured with BMSC-EVs (Fig. 4B). These results indicate that Smad 6 was somewhat associated with the BMSC-EVs-induced effects on the differentiation of NSCs.

### Smad 6 knockdown attenuated the BMSC-EVs-induced inhibitory effect on BMP signaling

To demonstrate that Smad 6 knockdown could affect BMSC-EVs-mediated BMP signaling, we detected the expression of Id2. Id2 is a member of the basic helix–loop–helix (bHLH) transcription factor family, which is upregulated by BMP/Smad 1/5/8 signaling [36], and is also considered to be an important transcription factor for BMP signaling in terms of differentiation of NSCs. Id2 mediates the differentiation of NSCs by the sequestering of oligodendrogenic transcription factors [37]. Studies have reported that an over-expression of Id2 is enhances astrocytic differentiation, leading to an increase in astrocytes and a reduction in neurons and oligodendrocytes [37, 38]. Therefore, we investigated whether the alteration in the differentiation of NSCs was associated with BMP signaling by evaluating Id2 expression.

As expected, the BMSC-EVs treatment markedly reduced Id2 expression in NSCs in the presence of BMP4. Although Id2 expression in Smad 6 knockdown NSCs that received BMSC-EVs was higher than that in the control groups, it was reduced as compared to the BMSC-EVs groups (Fig. 4C). This indicated that the BMSC-EVs-induced effect on Id2 expression was partly attenuated by Smad 6 knockdown. In summary, the BMSC-EVs-induced upregulation of Smad 6 inhibited the astrocytic differentiation of NSCs by repressing the BMP signaling pathway.

### TGF-β secreted from BMSCs upregulates the expression of Smad 6 in the later period of SCI

**Fig. 5:**
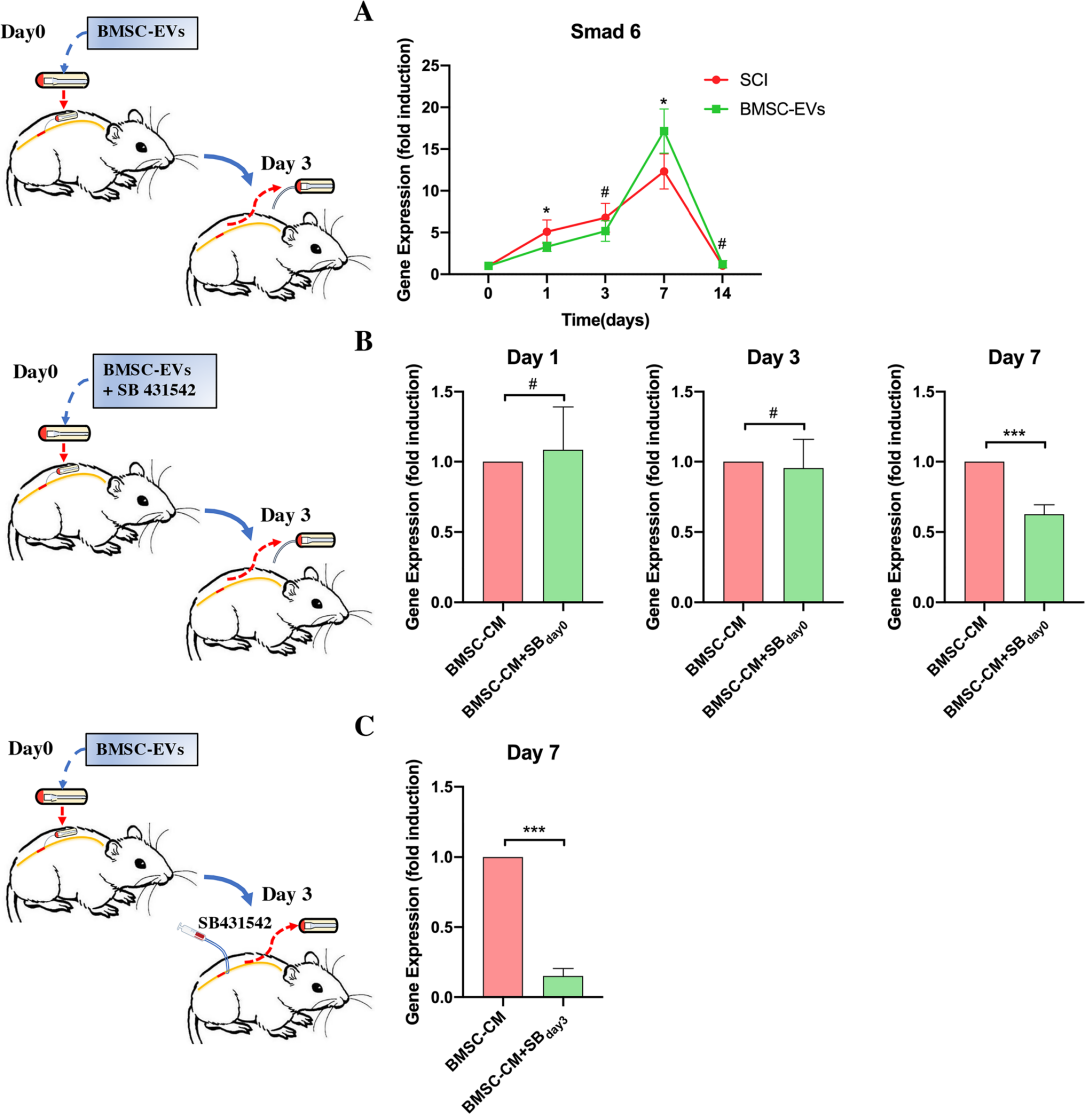
BMSC-EVs upregulated Smad 6 expression in the later phase of SCI. **A.** A pump containing DMEM/F12 (control SCI groups) or BMSC-EVs was implanted in SCI rats for 3 days. The results showed that the BMSC-EVs treatment reduced Smad 6 expression on day 1 and significantly increased Smad 6 expression on day 7 post SCI (*n* = 5). **B.** The SB_day0_ rats were treated with BMSC-EVs and SB431542 by a pump immediately following the SCI, and the results showed that, as compared to the control SCI rats, the expression of Smad 6 on days 1 and 3 post SCI was not altered by the early treatment with SB431542. However, the expression of Smad 6 on day 7 post SCI could be reduced by both the early and later treatment with SB431542 (*n* = 5). **C.** The SB_day3_ rats were first treated with BMSC-EVs by a pump for 3 days. When the pump was removed on day 3 post injury, SB 431,542 was injected through the soft catheter that connected the pump and the dura. The results showed that the expression of Smad 6 was reduced markedly on day 7 post injury (*n* = 5). (Student’s t-test was used for comparisons, ^*^p < 0.05, ^**^p < 0.01, ^***^p < 0.001, ^#^*p* > 0.05)

To identify whether BMSC-EVs were able to mediate Smad 6 expression in vivo, we assessed the expression of Smad 6 at different time points in SCI rats in groups that either had or had not received the BMSC-EVs treatment. Compared to SCI rats, those treated with BMSC-EVs exhibited decreased the Smad 6 expression in the early phase (days 1 and 4) and markedly increased the Smad 6 expression in the later phase (day 7) following the SCI (Fig. 5A). To further conform whether this BMSC-EVs-induced Smad 6 expression was associated with TGF-β, we added the SB 431,542 to the BMSC-EVs-treated rats on day 0 (together with BMSC-EVs treatment immediately following SCI) and on day 3 respectively. The results showed that the addition of SB431542 to the BMSC-EVs-treated SCI rats on day 0 did not significantly reduce Smad 6 expression in the early period of SCI (days 1 and 3) (*p* > 0.05). However, the day 0 addition of SB 431,542 mildly reduced Smad 6 expression in the later phase of SCI (day 7 post-injury; *p* < 0.05) (Fig. 5B). In contrast, the day 3 addition of SB 431,542 significantly reduced Smad 6 expression on day 7 post-injury (*p* < 0.05) as compared to rats that were treated only with BMSC-EVs (Fig. 5C). These results indicate that the expression of Smad 6 in the later period of SCI (day 7) is closely related to the BMSC-EVs treatment.

### A SB431542 treatment in a different phase of the SCI resulted in a distinct outcome

**Fig. 6:**
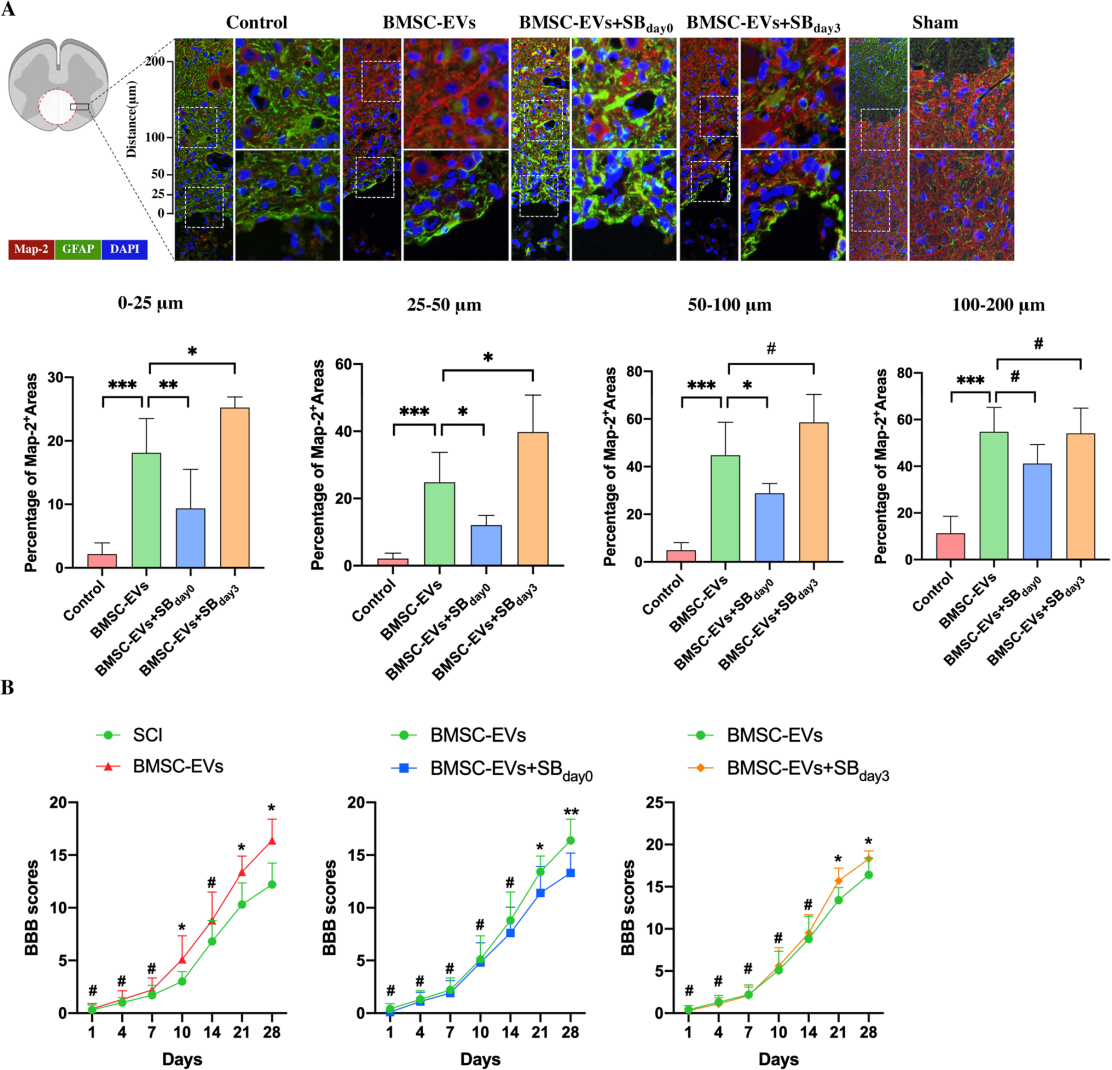
Inhibiting TGF-β in a different phase of SCI led to a distinct outcome. The SCI rats were treated with DMEM/F12 (control SCI groups), BMSC-EVs + SB 431,542 for 3 days (from the time points of injury to day 3 post injury, SB_day0_ groups), and BMSC-EVs for 3 days + SB 431,542 on day 3 post injury (SB_day3_ groups). Tissue immunofluorescence and BBB scores were then used to evaluate the histology of the injured lesions and the neurological functional outcome, respectively. **A.** The area occupied by Map-2-positive neurons and GFAP-positive scar-forming astrocytes within 200 μm from the edge of the cavity 4 weeks post SCI. Compared to the SCI rats, the BMSC-EVs-treated rats had more areas with neurons around the cavity. However, the SB 431,542 and BMSC-EVs treatment (SB_day0_ groups) somewhat reduced the Mpa-2^+^ areas around the cavity; more astrocytic scar tissue surrounding the cavity was noted in this group. Conversely, the area with neurons around the cavity was increased owing to the later treatment with SB431542 (SB_day3_ groups) (*n* = 5, student’s t-test was used for comparisons). **B.** BBB scores were in accordance with the spinal cord histology. Compared to the control SCI rats, the BMSC-EVs-treated rats had significantly improved neurological function outcomes. However, the early addition of SB 431,542 (SB_day0_ groups) worsened the neurological function outcome as compared to the rats that received only BMSC-EVs. In contrast, the later addition of SB 431,542(SB_day3_ groups) resulted in a better outcome than that seen in the BMSC-EVs rats (*n* = 10, Mann–Whitney U test was used for comparisons). ^*^p < 0.05, ^**^p < 0.01, ^***^p < 0.001, ^#^*p* > 0.05; SB_day0_, SB431542 treatment immediately following the SCI; SB_day3_, SB431542 treatment at day 3 following the onset of the SCI

To further evaluate the relationship between the expression of Smad 6 and neurological outcomes in SCI rats, we treated SCI rats with SB431542 in different phases (day 0 and day 3 post-injury) in the presence of BMSC-EVs. Immunostaining at week 4 following injury was used to explore the expression of GFAP and Map-2, revealing the number of astrocytes and neurons, respectively, in the injured lesion site. BBB scores were used to assess the neurological outcomes of SCI rats at different time points. The histological results showed that a large number of GFAP^+^ astrocytes surrounded the cavity that comprised a scar boundary in the control SCI rats.; few Map-2^+^ neurons were found there (Fig. 6A). Conversely, clear neurite outgrowth and extension into the scar tissues and a thin scar boundary were found in rats that received the BMSC-EVs treatment (Fig. 6A).

Based on the in vitro results, blocking TGF-β signaling could promote the generation of neurons in injured lesions. However, the addition of SB431542 to BMSC-EVs did not increase the number of neurons, but rather repressed the generation of Map-2^+^ neurons and their neurite outgrowth into the scar boundary, as compared to rats that received only the BMSC-EVs treatment (Fig. 6A). In addition, the subsequent treatment with SB431542 increased the Map-2 expression around the cavity. SB431542 treatments, which were administered later in the process, appeared to provide a better neurological functional outcome (as compared to rats that received the BMSC-EVs treatment), and SB431542 treatments that were administered earlier appeared to slightly attenuate the BMSC-EVs-induced improvements in SCI rats (Fig. 6B), which was consistent with the histological results.

**Fig. 7:**
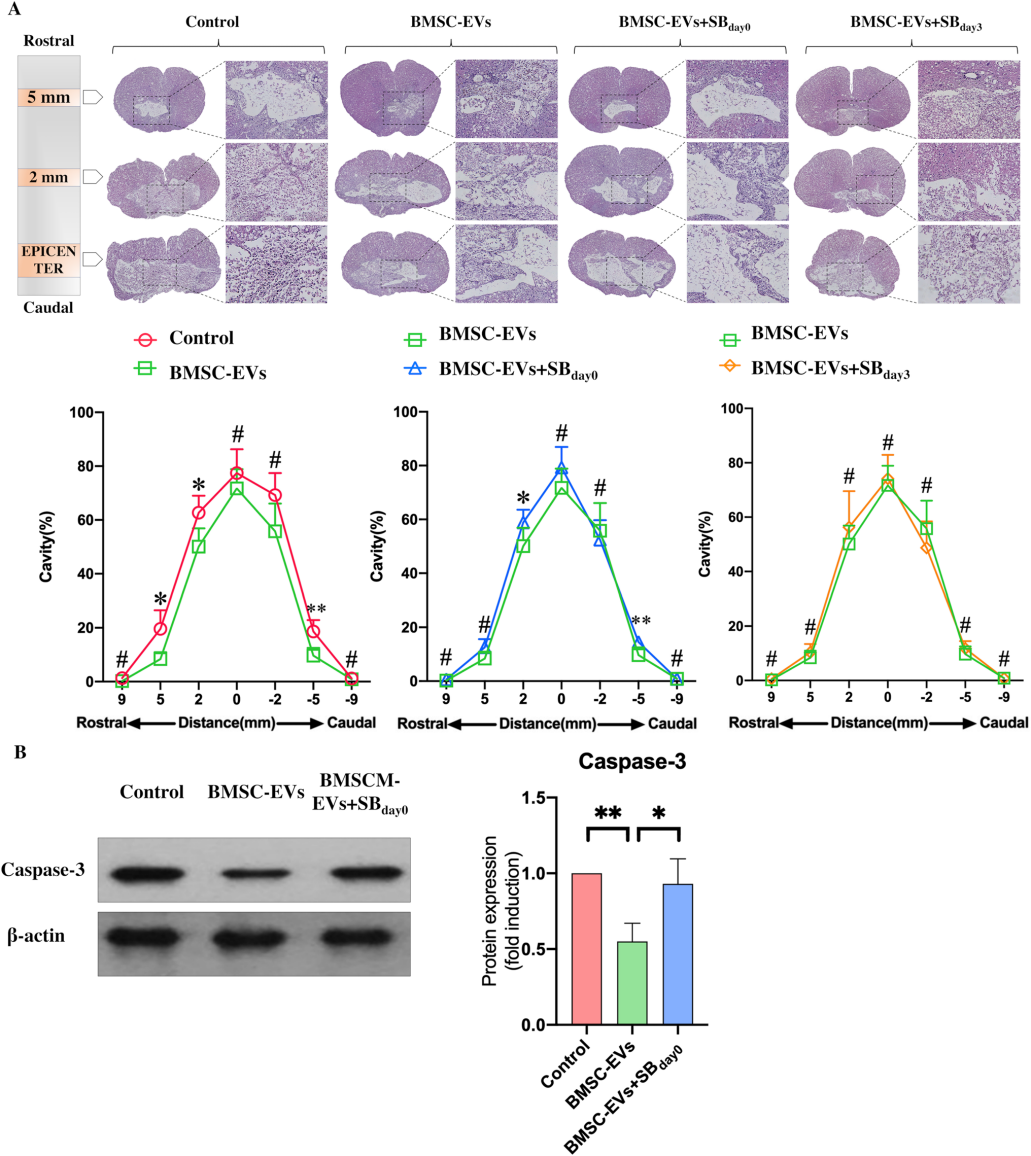
Inhibiting TGF-β in the early phase of SCI increased the cavity volume and the expression of Caspase-3. **A.** Hematoxylin–eosin staining was used to assess the cavity volume 4 weeks post injury. The results showed that the BMSC-EVs treatment markedly reduced the cavity volume of the injured lesion. The early addition of SB 431,542(SB_day0_ groups) to BMSC-EVs-treated-rats increased cavity volume. Conversely, no alteration in volume was induced by the later SB431542 treatment (SB_day3_ groups) (*n* = 5). **B.** The expression of Caspase-3 24 h post injury was used to evaluate the apoptosis in the injured lesion, which showed that the BMSC-EVs treatment reduced the expression of Caspase-3. However, these BMSC-EVs-induced effects were partly abolished by the early addition of SB 431,542 (*n* = 3). Student’s t-test was used for comparisons, ^*^p < 0.05, ^**^p < 0.01, ^***^p < 0.001, ^#^*p* > 0.05

TGF-β is considered an anti-inflammatory cytokine; although the inhibition of TGF-β was able to promote the generation of neurons, the inhibition of TGF-β in the early phase had the potential to lead to destructive inflammation, resulting in increased apoptosis of neurons at the adjacent injury lesion. This result could explain why the early addition of SB431542 reduced rather than promoted the generation of neurons around the cavity. To confirm this hypothesis, we assessed the cavity volume at 4 weeks post injury (Fig. 7A) and the expression of apoptosis protein (Caspase-3) at 24 h post-injury (Fig. 7B). The results showed that BMSC-EVs-treated rats experienced significant decreases in cavity volume and Caspase-3 expression. Moreover, the early addition of SB431542 to the BMSC-EVs treatment somewhat reduced the BMSC-EVs-induced effects. However, the subsequent addition of SB 431,542 did not increase the cavity volume as compared to the rats that received the BMSC-EVs treatment.

### The expression of Smad 6 was affected by the addition of IL-6 or BMP4

**Fig. 8:**
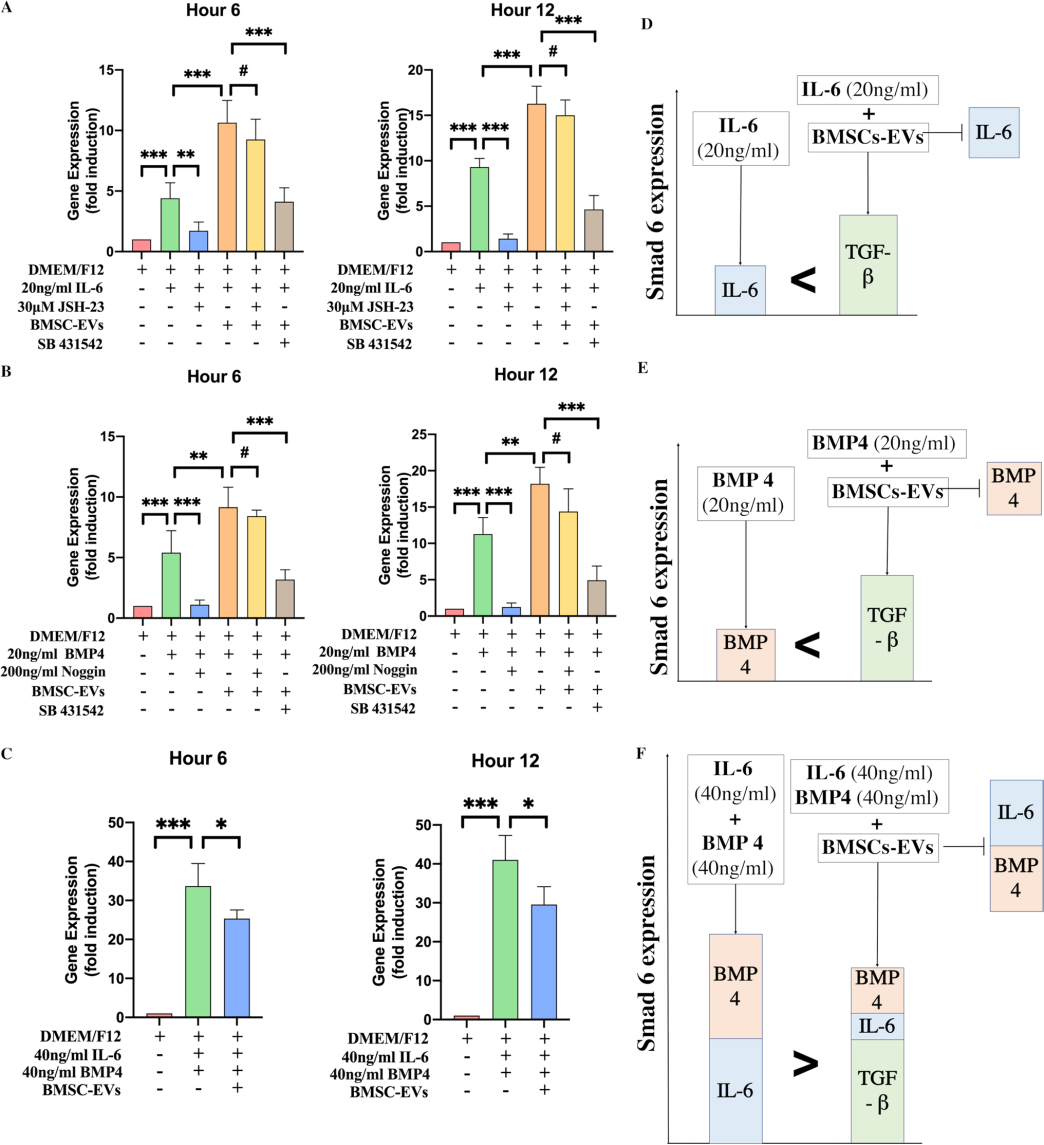
Expression of Smad 6 in different groups. **A.** The NSCs were treated with 20 ng/mL IL-6, with or without 30 μM JSH-23. Next, the BMSC-EVs were added to NSCs in the presence of IL-6 in combinations with or without SB 431,542. RT-PCR was performed to analyze the expression of Smad 6 in NSCs at 6 h and 12 h; the results showed that the expression of Smad 6 was upregulated by the addition of IL-6 and that this upregulation could be completely abolished by the addition of JSH-23 (*n* = 3). The addition of BMSC-EVs could upregulate the Smad 6 expression in the presence of IL-6 (*n* = 3), and this BMSC-EVs-related upregulation was inhibited by the SB 431,542 treatment (*n* = 3). **B.** Similarly to IL-6, the BMSC-EVs could upregulate the Smad 6 expression in NSCs in the presence of BMP4 (*n* = 3), and this upregulation was abolished by the SB 431,542 treatment (*n* = 3). **C.** The NSCs were co-cultured with 40 ng/mL IL-6 and 40 ng/mL BMP4 in combination with or without BMSC-EVs; the results revealed that the addition of BMSC-EVs downregulated rather than upregulated the expression of Smad 6 in NSCs in the presence of a high dose of IL-6 and BMP4 (*n* = 3). (Student’s t-test was used for comparisons, ^*^p < 0.05, ^**^p < 0.01, ^***^p < 0.001, ^#^*p* > 0.05). **D, E, F.** Schematic representation of the mediating effects of IL-6, BMP4, and BMSC-EVs on Smad 6 expression

The indication that the BMSC-EVs treatment did not increase Smad 6 expression in the early phase of SCI rats as it had increased Smad 6 expression in vitro was an unexpected result. As previously mentioned, Smad 6 expression is not only mediated by the TGF-β, but also by the BMPs and NF-κB signaling, which could be repressed by BMSCs. Therefore, we hypothesized that it was BMPs and NF-κB signaling, inhibited by the BMSC-EVs, which neutralized the increase in Smad 6 expression induced by TGF-β in the early phase of injury. To confirm this, we first added either IL-6 or BMP4 to NSCs either with or without the NF-κB inhibitor (JSH-23) or BMP4 inhibitor (Noggin) and examined the resulting expression of Smad 6 in NSCs. As expected, both IL-6 and BMP4 were able to increase the Smad 6 expression. Moreover, these IL-6-induced or BMP4-induced effects were abolished by the addition of their inhibitors (Fig. 8A, B).

Surprisingly, the expression of Smad 6 in NSCs was not reduced by the addition of BMSC-EVs in the presence of IL-6 or BMP4; instead, the BMSC-EVs increased Smad 6 expression in NSCs as compared to the IL-6- or BMP4-treated NSCs (Fig. 8A, B). This might be because that TGF-β had a stronger effect on increasing Smad 6 expression in NSCs than either IL-6 or BMP4. Therefore, although BMSC-EVs abolished the IL-6 or BMP4-induced increase in the expression of Smad 6, TGF-β (which was secreted by BMSCs) was still able to increase Smad 6 expression. To prove this, we added either the NF-κB or BMP4 inhibitor to NSCs, which did not reduce the expression of Smad 6, while the addition of SB 431,542 significantly reduced Smad 6 expression in NSCs cultured with BMSC-EVs, and either IL-6 or BMP4 (Fig. 8A, B). These results indicated that relatively low doses of IL-6 or BMP4 could be nearly completely inhibited by the BMSC-EVs, and as the low doses of IL-6 or BMP4 had a weaker effect on the upregulation of Smad 6 expression than TGF-β did, the addition of BMSC-EVs was still able to upregulate Smad 6 expression in such a manner that IL-6 and BMP4-related signaling pathways were abolished (Fig. 8D, E).

To further imitate the situation of the early phase of SCI, we increased the doses of IL-6 and BMP4 and added them to NSCs. This was done in an attempt to generate a condition in which the IL-6 or BMP4-induced upregulation of Smad 6 expression could be significantly but not completely abolished by BMSC-EVs, so that the decrease of IL-6 and BMP4 might lead to a marked reduction in the Smad 6 expression. This decreased in IL-6/BMP 4 had an effect on the downregulation of Smad 6 expression, which was stronger than the TGF-β-induced effect on the upregulation of Smad 6 expression. These results showed that the co-addition of high doses of IL-6 and BMP4 markedly elevated Smad 6 expression in NSCs and, as expected, the addition of BMSC-EVs resulted in a reduction rather than an increase in Smad 6 expression in NSCs (Fig. 8C). These in vitro results explain why the addition of BMSC-EVs did not increase Smad 6 expression in the early phase of SCI.

## Discussion

**Fig. 9:**
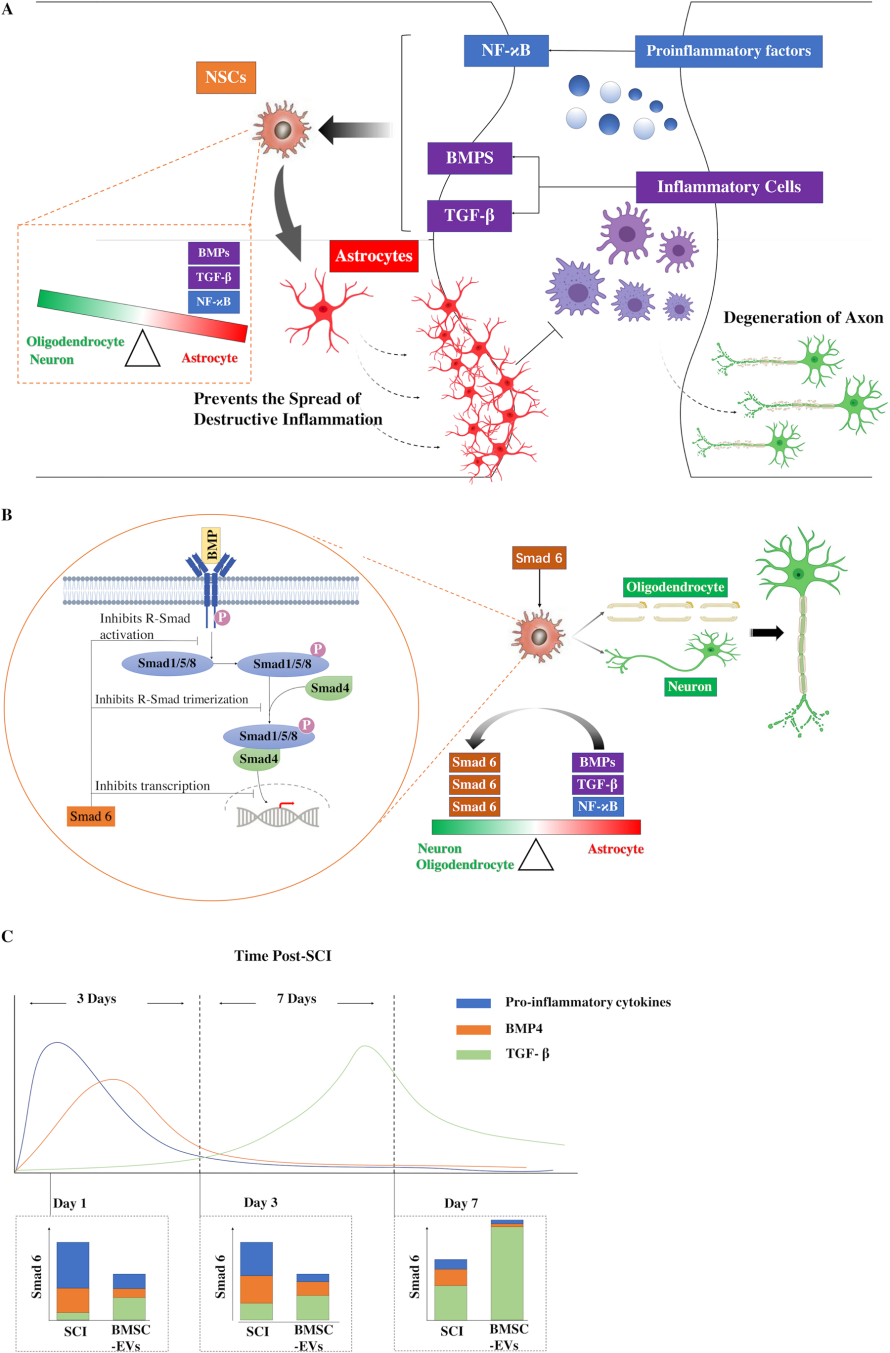
Various factors accumulated in the injury lesion and induced the differentiation of NSCs into astrocytes, which contributed to the prevention of the spread of destructive inflammation and protected the adjacent neural cells from apoptosis (A). Meanwhile, these factors also upregulated the expression of Smad 6, which acted as a negative feedback regulator of the astrocytic differentiation and prevented the over-formation of glial scars(B). **C.** Schematic representation of the mechanism that mediates the Smad 6 expression in different phases following SCI with or without the BMSC-EVs treatment

Following SCI, endogenous NSCs around the injury lesion are activated and rapidly migrate to the lesion site. However, in this unfavorable microenvironment, most of these activated NSCs do not differentiate into neurons or axons; instead, they differentiate into astrocytes that form a glial scar around the injured cavity [3]. BMPs have been reported to play a key role in the promotion of gliosis [39]; their levels increase and they accumulate in the injury lesion, contributing to the glial differentiation of endogenous NSCs following SCI [5]. During the acute phase of the injury, the scar boundary formed around the injured core is crucial for the prevention of the spread of early inflammation and the protection of the adjacent surviving neural cells from destructive inflammation (Fig. 9). The glial scar has long been thought to be the main reason for the failure of the neurons and axons to regenerate and remodel [9, 40]. However, recent studies have shown that preventing glial scar formation in SCI does not result in greater axon regeneration or better neurological recovery in SCI [9, 41]. With appropriate growth factor supplementation, axons are able to regrow along the scar boundary after CNS injury [42], and SCI models have also demonstrated that axon regeneration can be improved by grafting astrocytes [43–45]. Although astrocytes may aid in axon regeneration, the over-generation of glial scars is still considered to hinder the remodeling or regeneration of axons, especially in the chronic phase of SCI. Therefore, the regulation of astrocytes and scar formation is critical for neurological improvement in SCI.

BMP signaling has been shown to play an important role in the formation of astrocyte scars following the onset of SCI. The upregulation of BMPs in the acute phase of SCI could aid in the rapid formation of the scar boundary [5], which is helpful for limiting inflammation and protecting the surviving adjacent cells [6–8, 46]. However, the expression levels of these factors remain high after the early phase of SCI, resulting in the over-generation of astrocyte scars around the injured cores. Therefore, I-Smads act as a negative feedback regulator of BMP signaling, which might be effective in limiting the over-expression of astrocytes. The results of the present study showed that the knockdown of Smad 6 expression in NCSs resulted in an increase in the proportion of astrocytes, a reduction in the number of neurons, and a decrease in the expression of Id2, indicating that Smad 6 was able to antagonize the BMP-induced effect and promote the differentiation of NSCs to neurons. Therefore, balancing between R-Smads and I-Smads is critical for mediating scar formation.

It is generally thought that MSCs exert their biological effects in different models by secreting a large variety of factors and molecules [47–50]. In SCI rats, MSCs were able to improve neurological outcomes by promoting the regeneration of neurons and axon regrowth [51–53] through the inhibition of BMP/Smad signaling [5, 27]. The present study found that the addition of BMSC-EVs to NSCs increased the expression of Smad 6, and the BMSC-EVs-induced effect on Id2 expression and differentiation of NSCs was partially abolished by Smad 6 knockdown, which suggests that BMSCs mediated the differentiation of NSCs not only by inhibiting BMP/Smad signaling but also in part by upregulating the expression of Smad 6.

HGF, which is released by MSCs, proved to be a key factor in the BMSC-EVs-associated mediation of BMP/R-Smad signaling [27, 54, 55]. HGF was previously shown to be unable to affect the expression of I-Smads[56], and the present study offered evidence that TGF-β might be crucial in the upregulation of Smad 6 in NSCs. First, in accordance with previous studies [29, 57], TGF-β was found in the secreted medium, proving that it was released by BMSCs. Second, the BMSC-EVs-induced upregulation of Smad 6 was abolished by the addition of the TGF-β inhibitor SB431542. Finally, the SB431542 and BMSC-EVs treatment reduced reduced Smad 6 expression in the later phase of SCI.

The results revealed that BMSC-EVs were able to increase the expression of Smad 6 in NSCs in vitro. However, the in vivo results showed that the BMSC-EVs treatment did not affect the expression of Smad 6 in SCI rats. In the early period of SCI, the expression of Smad 6 was reduced by the BMSC-EVs treatment, which may be explained by the fact that Smad 6 expression is not regulated solely by TGF-β, but BMP signaling and inflammatory cytokines are also involved. In the early phase of SCI, inflammatory cells are recruited to the injured lesion, which induces a rapid increase in pro-inflammatory cytokines [58, 59] and BMP expression [5]. MSCs have been shown to repress both the production of pro-inflammatory cytokines and BMP signaling [5, 27, 32], and it is thus possible that the BMSC-EVs-induced repression of pro-inflammatory cytokines and BMP signaling led to a reduction in Smad 6 expression in the early phase of SCI. This could also explain why administering the SB431542 treatment immediately following SCI did not markedly alter Smad 6 expression in the early period. We imitated such a condition in vitro by adding high doses of IL-6 and BMP4 to NSCs. The addition of BMSC-EVs reduced the Smad 6 expression in this case, indicating that the IL-6- or BMP 4-related upregulated effects on Smad 6 expression were inhibited. This reduction led to a stronger decrease in Smad 6 expression than the TGF-β-induced increase in Smad 6 expression, resulting in a reduction of Smad 6 expression after the addition of BMSC-EVs in the presence of IL-6 and BMP4.

Although Smad 6 was not altered by the SB431542 treatment in the early period of SCI, it was significantly reduced by the SB431542 treatment in the later phase of SCI. TGF-β, which is produced by inflammatory cells, can repress the destructive inflammatory process, either by inhibiting the activation of NF-κB[60] or by directly suppressing T_H_1 cells [61]. TGF-β expression began to increase 24 h after SCI and reached a relatively high level after approximately 1 week [62]. In contrast, the expression of pro-inflammatory cytokines reached a high level in a short time; it gradually decreased after 3 days post injury and dropped to normal levels after 7–14 days[27, 32, 62]. Similarly, the levels of BMPs produced by inflammatory cells increased rapidly following the onset of SCI and then decreased gradually [5, 27]. Therefore, the expression of pro-inflammatory cytokines and BMPs stabilizes at a normal level, while TGF-β expression remains at a high level in the later phase of SCI, indicating that the relatively high expression of TGF-β, as compared to pro-inflammatory cytokines and BMPs, plays a key role in the upregulation of Smad 6. This explains why the expression of Smad 6 in the later phase of SCI was reduced by the SB431542 treatment. This result was in accordance with the in vitro data: the addition of BMSC-EVs increased the Smad 6 expression in the presence of a relatively low doses of IL-6 or BMP4. This indicates that relatively low doses of IL-6 or BMP4 had a weaker mediating effect on the Smad 6 expression, although Smad 6 expression was inhibited by the addition of BMSC-EVs, and the TGF-β in BMSC-EVs was still able to upregulate the Smad 6 expression in NSCs.

Another notable result is that the addition of SB431542 to the BMSC-EVs treatment in rats at different phases of SCI generated distinct outcomes. Compared to the BMSC-EVs-treated rats, rats that received both SB431542 and BMSC-EVs immediately following the onset of SCI exhibited worse histology results and lower BBB scores. In contrast, rats that received BMSC-EVs in the early phase and SB431542 at day 3 post injury had a higher proportion of neurons and a thinner scar boundary around the injured lesion, as well as higher BBB scores. These results could be explained as follows: First, TGF-β acts as an anti-inflammatory cytokine. It plays an important role in mediating the inflammatory processes [63, 64]. The inhibition of TGF-β in the early phase led to the over-activation of inflammation-associated signaling, resulting in the apoptosis of neurons around the injury lesion. Second, TGF-β promotes gliosis in the CNS [65]. The early inhibition of TGF-β might lead to a dysfunction in the scar formation process, which would then attenuate the effect on the limitation of inflammation. These two points were confirmed by examining the cavity volume and the Caspase-3 expression. These two indices were increased by the early treatment with SB 431,542, indicating worse destructive inflammation at the lesion site. This explains why the early blocking of TGF-β induced worse histological results and neurological outcomes. Finally, the inflammation is mostly stable, and neurons begin to regenerate in the later phase of SCI; the inhibition of TGF-β may not cause an increase in inflammation during this phase. In contrast, TGF-β was able to promote neurogenesis in the injured lesions in the later phase of the injury. This explains why the addition of SB431542 increased neuronal expression and promoted a functional neurological outcome in the later phase.

This study provides evidence that Smad 6 is able to prevent NSCs from over-differentiating into astrocytes in vitro. However, due to the lack of a direct inhibitor of Smad 6 and Smad 6 knockout in mice, which may possibly affect CNS development, we could not directly counter Smad 6 effects in SCI rats. Hence, we indirectly downregulated Smad 6 expression in vivo by inhibiting TGF-β signaling, which mediates NSC differentiation. This, combined with the in vitro results, indirectly highlights the role of Smad 6 in mediating the differentiation of NSCs in SCI rats, although further in vivo studies are required.

In conclusion, BMSC-EVs upregulated Smad 6 expression through TGF-β. Smad 6 acted as a negative feedback regulator that inhibited BMP/Smad 1/5/8 signaling and promoted the differentiation of NSCs into neurons. These results indicate that Smad 6 could be a potential therapeutic target for the treatment of spinal cord injuries.

## Data Availability

The datasets used and/or analyzed during the current study are available from the corresponding author upon reasonable request.

## Abbreviations

SCI: Spinal cord injury
MSCs: Mesenchymal stem cells
BMSCs: Bone marrow-derived mesenchymal stem cells
NSCs: Neural stem cells
eNSCs: Endogenous neural stem cells
TGF-β: Transforming growth factor β
CNS: Central nervous system
EVs: Extracellular vesicles
BMPs: Bone morphogenetic proteins
GFAP: Glial fibrillary acidic protein
Map-2: Microtubule-associated protein 2
SB: SB-431542
